# Age and waist circumference as key determinants of postoperative thrombosis and squatting recovery after unicompartmental knee arthroplasty

**DOI:** 10.1007/s40520-025-02974-0

**Published:** 2025-03-19

**Authors:** Xuyang Cao, Mengsha Wang, Zizi Zhao, Taotao Kong

**Affiliations:** Department of Joint Orthopaedics, North China Medical Xingtai General Hospital, No. 202 Bayi Road, Xingtai, Hebei 054000 China

**Keywords:** Thrombosis, Squatting, Unicompartmental knee arthroplasty, Age, Osteoarthritis

## Abstract

**Background:**

Thrombosis is a common postoperative complication after unicompartmental knee arthroplasty (UKA), and the ability to squat is an important functional outcome reflecting recovery of knee function. This study aimed to investigate the factors influencing postoperative thrombosis as well as the ability to squat within 1 year after UKA.

**Methods:**

Data from UKA patients were retrospectively analyzed and grouped based on the occurrence of thrombosis (including deep vein thrombosis and superficial vein thrombosis) and the ability to squat within 1 year. Factors affecting thrombosis and squatting were compared and analyzed using multifactorial logistic regression.

**Results:**

Univariate analysis revealed that age (*P* = 0.014), pre-operative haemoglobin (*P* = 0.044), and gender (*P* = 0.047) were associated with thrombosis, while multifactorial analysis found age (*P* = 0.024) as the key factor. Regarding squatting ability, univariate analysis identified age (*P* = 0.018), body weight (*P* = 0.001), BMI (*P* = 0.001), waist circumference (*P* < 0.001), pre-operative VAS score (*P* = 0.002), and family living conditions (*P* = 0.019) as influencing factors, with multifactorial analysis identifying waist circumference (*P* = 0.002) as a significant factor.

**Conclusions:**

Older age increases the likelihood of thrombosis after UKA. Additionally, a larger waist circumference decreases the likelihood of squatting within 1 year after surgery.

## Introduction

Osteoarthritis (OA) of the knee is a significant public health challenge, with its burden increasing due to an aging population and rising risk factors such as obesity [1–3]. The demand for knee arthroplasty is expected to increase more than sixfold by 2030 [4]. Among patients with knee OA, degeneration and damage of the medial compartment cartilage often result in medial knee pain, which can progress to inversion deformity as the condition worsens [5]. While total knee arthroplasty (TKA) is a well-established surgical treatment, it has limitations, including longer recovery times and a higher risk of postoperative complications [6]. In recent years, unicompartmental knee arthroplasty (UKA) has gained attention due to its minimally invasive nature, faster recovery, and improved patient outcomes [7].

Venous thromboembolism (VTE), which includes deep vein thrombosis (DVT) and its potential complication, pulmonary embolism (PE), is a common postoperative complication of knee arthroplasty [8]. In the absence of thrombotic interventions, the incidence of venous thromboembolism after knee arthroplasty can reach 88% [9], and after antithrombotic treatment, the incidence is reduced to around 20% [10]. Additionally, superficial venous thrombosis (SVT) is another recognized postoperative concern that may affect recovery [11, 12]. Despite this, the factors influencing the incidence of thrombosis specifically after UKA remain unclear.

In addition to thrombosis, the recovery of joint function—particularly the ability to squat—serves as a key indicator of postoperative success and quality of life. The ability to squat is especially important for performing daily activities, such as sitting on low surfaces or picking up objects from the floor. However, the recovery of squatting function typically requires a longer rehabilitation period and is often a delayed milestone in the UKA recovery process. Currently, there is a lack of comprehensive studies investigating the factors influencing squatting ability within the first year after UKA [13].

Given that thrombosis and squatting recovery both significantly influence patient outcomes after UKA, this study aimed to evaluate these two aspects together. Thrombosis directly impacts the safety and overall recovery of patients, while squatting ability reflects functional outcomes and long-term satisfaction. By analyzing both thrombosis and squatting recovery, we sought to provide a more holistic understanding of the factors that influence both clinical safety and functional recovery after UKA. This approach also allowed us to identify potential interrelationships between these outcomes, offering valuable insights for optimizing postoperative rehabilitation and improving patient care.

In this study, we retrospectively analyzed the clinical data of 99 patients who underwent UKA, comparing differences in key factors between those with and without thrombosis (including both deep vein thrombosis and superficial vein thrombosis) and between those who were able to squat and those who were not within 1 year. We hypothesize that certain clinical factors influence both the incidence of thrombosis and the ability to squat, and aim to provide theoretical support for post-operative rehabilitation strategies following UKA.

## Materials and methods

### General data

The data of patients who underwent UKA surgery for medial compartment knee osteoarthritis collected from January 2018 to January 2020 at our institution were retrospectively analyzed. The patients were divided into two groups based on the presence of thrombosis (including DVT) and superficial vein thrombosis), with each group analyzed separately. A total of 99 cases were gathered. All patients were followed up for more than 1 year with a mean of (12.22 ± 7.54) months. The general data of the patients were analyzed, as well as the presence of thrombosis after UKA and the ability to squat at one year. The study protocol complied with the relevant requirements of the World Medical Association Declaration of Helsinki [14] and was approved by the Ethics Committee of our hospital.

### Inclusion criteria

Patients were included if they met the following criteria:

1. Diagnosed with anteromedial osteoarthritis based on clinical and radiographic findings.
2. Intact and functioning anterior cruciate ligament confirmed via physical examination or imaging.
3. Knee range of motion > 90°.
4. Varus deformity < 15°, passively correctable to neutral alignment.
5. Pain localized to the medial compartment of the knee.
6. Availability of complete clinical data and follow-up records.
7. Provided written informed consent prior to surgery.

### Exclusion criteria

Patients were excluded if they met any of the following criteria:

1. History of prior high tibial osteotomy or other significant knee surgeries.
2. History of inflammatory or infectious arthritis, including rheumatoid arthritis, traumatic arthritis, gouty arthritis, or tuberculous arthritis, as these conditions are contraindications for unicompartmental knee arthroplasty (UKA).
3. Severe patellofemoral arthritis or patellar subluxation.
4. Presence of severe systemic diseases (e.g., cardiovascular, respiratory, or renal conditions) that rendered the patient unable to tolerate surgery.

### Surgical approach

All patienrs underwent the same anesthesia method. Upon entering the operating room, patients were monitored for routine vital signs, and a peripheral intravenous line was established. Combined spinal-epidural anesthesia was administered with the patient in a lateral decubitus position (affected side down). A subarachnoid puncture was performed at the L2-3 or L3-4 interspace, and a total of 3 mL of anesthetic solution was injected, consisting of 1 mL of 10% glucose and 2 mL of 0.5% bupivacaine. The anesthetic level was achieved up to the T8–T10 dermatomes. Following successful anaesthesia, the patient is placed in the supine position, with routine iodine and alcohol skin disinfection and sterile towels. The knee is flexed at 90°, a longitudinal incision is made from the medial aspect of the superior pole of the patella to the medial aspect of the tibial tuberosity, measuring approximately 7 cm in length, the soft tissues around the joint are loosened, the bone and meniscus are removed, a tibial guide is used for tibial osteotomy and trial moulding, a suitable femoral prosthesis model is selected, the cartilage on the surface of the femur is removed, a trial mould of the tibia and femur is fitted and the joint space and stability are checked. The joint cavity was flushed, the platform unicondylar prosthesis and liner were fixed, the knee joint was moved to check knee stability and patellar dislocation. After repeated flushing the periarticular cocktail was analgesically placed, drains were placed and the incision was closed layer by layer and then wrapped with a sterile dressing elastic band.

Postoperative antithrombotic therapy with low molecular heparin calcium injection (100 units/kg) and additional physical anticoagulation with air pressure. Postoperative follow-up included venous ultrasound of both lower extremities on postoperative day 7 to diagnose thrombosis. This timing was chosen because it represents a relatively high-risk period for deep vein thrombosis (DVT) formation following knee arthroplasty [15].

### Postoperative follow-up and rehabilitation exercises (7 Days)

On the day of surgery, patients were guided to perform isometric quadriceps contractions. After removal of urinary and drainage catheters, they were encouraged and assisted to get out of bed for appropriate activities, primarily focusing on ankle pump exercises to reduce the risk of deep vein thrombosis. On postoperative day 1, patients were instructed to continue with quadriceps and ankle pump exercises, as well as engage in assisted walking and standing exercises using a walker. Passive knee extension and flexion exercises were also introduced. By postoperative day 3, patients began knee extension training, active bedside knee flexion exercises, thigh-hugging active knee flexion movements, and supine straight-leg raises. Between postoperative days 5 and 6, the aforementioned exercises were repeated, with the addition of stair-climbing exercises as appropriate. Before discharge, an individualized rehabilitation plan was developed for each patient, with instructions to adhere to functional exercises to support recovery.

### Squatting function evaluation

The squatting function of patients was assessed using the 7th item of the Oxford Knee Score (OKS) [16], which evaluates the ability to squat. The scoring criteria were as follows: a score of 1 indicated the ability to squat easily, while scores of 2, 3, and 4 represented mild, moderate, and severe difficulty in squatting, respectively. A score of 5 indicated an inability to squat, with higher scores reflecting poorer squatting function of the knee joint. The evaluations were conducted by experienced nursing staff from our institution who had undergone specific training in the use of the OKS for assessing squatting function, ensuring accuracy and consistency in the assessments.

### Statistical analysis

Data were expressed as frequency or mean ± standard error of the mean as appropriate.

Univariate logistic regression, chi square tests, or Fisher’s exact tests were used for univariate analysis as appropriate. Variables reaching *P* < 0.10 in univariate analysis were included in multivariate analysis. *P* < 0.05 was used to indicate statistical significance for all comparisons. Multivariate analysis was performed with logistic regression. Statistical analysis was performed using SPSS statistical software (Version 23, IBM corp.)

## Results

A total of 99 cases of UKA were collated and analyzed, of which 31 (31.31%) were male and 68 (68.69%) were female; 54 (54.55%) cases with hypertension and 4 (4.04%) with diabetes mellitus; 91 of all cases did not need to go up and down stairs at home and 8 needed to go up and down stairs at home. The mean age of the patients was 63.27 ± 6.87 years; the mean operating time was 52.38 ± 20.32 min; the pre-operative haemoglobin was 135.04 ± 13.53 g/L; the post-operative platelets were 207.56 ± 52.28 × 10^9/L; and the pre-operative VAS score was 3.07 ± 0.26. General data details are presented in Tables 1 and 2.

**Table 1:** General quantitative data of patients

Item	Number	Mean	Standard deviation	Min	Max	Missing number
Age (year)	99	62.27	6.87	48.00	80.00	0
Height (m)	99	1.62	0.06	1.50	1.75	0
Body weight (kg)	99	70.29	9.91	50.00	95.00	0
BMI	99	26.62	3.14	19.50	34.90	0
Leg circumference (cm)	99	55.52	6.84	41.00	68.00	0
Waist circumference (cm)	99	95.71	13.60	69.00	130.00	0
Duration of illness 1(year)	99	4.88	5.06	0.08	30.00	0
Duration of illness 2(years)	92	1.16	1.46	0.01	10.00	7
Tourniquet application time(min)	99	35.67	14.89	10.00	70.00	0
Operating time(min)	99	52.38	20.32	21.00	104.00	0
Pre-operative haemoglobin (g/L)	99	135.04	13.53	87.00	180.00	0
Pre-operative platelets (10^9/L)	99	246.38	61.09	106.00	425.00	0
Post-operative haemoglobin (g/L)	99	120.97	12.72	74.00	149.00	0
Post-operative platelets (10^9/L)	99	207.56	52.28	91.00	350.00	0
Intraoperative bleeding (ml)	98	106.12	32.32	50.00	200.00	1
Pre-operative VAS score	99	3.07	0.26	3.00	4.00	0

BMI, body mass index. VAS, visual analogue scale

**Table 2:** General count data of patients

Variable	Variable level	Frequency (%)	Cumulative frequency (%)
Gender	Male	31 (31.31)	31 (31.31)
	Female	68 (68.69)	99 (100.0)
Family living conditions	No need to go up or down the stairs	91 (91.92)	91 (91.92)
	To go up and down stairs	8 (8.08)	99 (100.0)
Occupation	Farmer	96 (96.97)	96 (96.97)
	Worker	3 (3.03)	99 (100.0)
Previous knee surgery	No	90 (90.91)	90 (90.91)
	Yes	9 (9.09)	99 (100.0)
High blood pressure	No	45 (45.45)	45 (45.45)
	Yes	54 (54.55)	99 (100.0)
Diabetes mellitus	No	95 (95.96)	95 (95.96)
	Yes	4 (4.04)	99 (100.0)
Arthritis classification	Class III	95 (95.96)	95 (95.96)
	Class IV	4 (4.04)	99 (100.0)
Type of prosthesis used	Extra Small	25 (25.51)	25 (25.51)
	Large	3 (3.06)	28 (28.57)
	Small	46 (46.94)	74 (75.51)
	Medium	24 (24.49)	98 (100.0)

In order to analyze the factors influencing thrombosis after UKA, the cases were divided into two groups: with or without thrombosis. one out of the 99 patients had missing data on thrombosis, while 29 (29.59%) of the remaining 98 cases had thrombosis after surgery and 69 (70.41%) had no thrombosis after surgery (Table 3).

**Table 3:** Count data for postoperative squatting (within 1 year) and thrombosis

Variable	Variable level	Frequency (%)	Cumulative frequency (%)
Post-operative thrombosis	No	69 (70.41)	69 (70.41)
	Yes	29 (29.59)	98 (100.0)
Squatting within 1 year of surgery	No	41 (41.41)	41 (41.41)
	Yes	58 (58.59)	99 (100.0)

General information was compared between the two groups by univariate analysis. Three factors, age (*P* = 0.014), pre-operative haemoglobin (*P* = 0.044), and gender (*P* = 0.047), were found to be significantly different between the two groups (Tables 4 and 5). For multivariate analysis of factors affecting post-operative thrombosis in UKA, we included all factors with a *P* < 0.1, therefore, waist circumference (*P* = 0.072) was additionally included in the multivariate logistic regression analysis, while weight and leg circumference were not included because they were factors that similar to waist circumference (Table 4). The results of the multifactorial logistic regression analysis showed that age was a factor affecting thrombosis after UKA (*P* = 0.024), with older age being more likely to cause thrombosis after surgery (Table 6).

**Table 4:** Univariate analysis of postoperative thrombosis (quantitative data)

Variables	Group	Sample size(Missing number)	Mean ± standard deviation	Median (interquartile spacing)	Statistic (T or Z)	*P*
Age (years)	No	69 (0)	61.25 ± 6.70	61.00 (56.00, 67.00)	-2.514	0.014
	Yes	29 (0)	64.97 ± 6.65	64.00 (60.00, 68.00)		
	Difference (row 1 - row 2)		-3.72	95%CI (-6.66,-0.78)		
Height (m)	No	69 (0)	1.63 ± 0.07	1.60 (1.58, 1.68)	-1.095	0.274
	Yes	29 (0)	1.61 ± 0.04	1.60 (1.59, 1.63)		
	Difference (row 1 - row 2)		0.02	95%CI (0.00,0.04)		
Body weight (kg)	No	69 (0)	71.35 ± 10.48	70.00 (65.00,80.00)	-1.739	0.082
	Yes	29 (0)	67.48 ± 7.94	67.50 (64.00, 70.00)		
	Difference (row 1 - row 2)		3.87	95%CI (-0.44, 8.17)		
BMI	No	69 (0)	26.85 ± 3.26	26.60 (24.50, 28.70)	1.247	0.216
	Yes	29 (0)	25.99 ± 2.81	26.00 (24.50, 27.30)		
	Difference (row 1 - row 2)		0.86	95%CI (-0.51, 2.24)		
Leg circumference (cm)	No	69 (0)	56.30 ± 6.91	58.00 (52.00, 61.00)	-1.729	0.084
	Yes	29 (0)	53.55 ± 6.48	55.00 (49.00, 58.00)		
	Difference (row 1 - row 2)		2.75	95%CI (-0.23, 5.74)		
Waist circumference (cm)	No	69 (0)	97.23 ± 14.06	101.00 (86.00, 108.00)	1.822	0.072
	Yes	29 (0)	91.79 ± 11.99	92.00 (82.00,102.00)		
	Difference (row 1 - row 2)		5.44	95%CI (-0.49, 11.36)		
Duration of illness 1(year)	No	69 (0)	4.69 ± 4.66	3.00 (2.00, 5.00)	0.215	0.830
	Yes	29 (0)	5.44 ± 6.02	4.00 (1.00, 8.00)		
	Difference (row 1 - row 2)		-0.74	95%CI (-3.26, 1.78)		
Duration of illness 2(yrs)	No	67 (2)	1.21 ± 1.51	1.00 (0.25, 2.00)	-0.642	0.521
	Yes	24 (5)	1.06 ± 1.32	0.50 (0.25, 1.00)		
	Difference (row 1 - row 2)		0.16	95%CI (-0.54, 0.85)		
Tourniquet application time (min)	No	69 (0)	35.22 ± 14.68	30.00 (24.00, 45.00)	0.66	0.509
	Yes	29 (0)	37.28 ± 15.52	40.00 (23.00, 50.00)		
	Difference (row 1 - row 2)		-2.06	95%CI (-8.62, 4.50)		
Operating time (min)	No	69 (0)	52.13 ± 20.15	47.00 (39.00, 66.00)	0.265	0.791
	Yes	29 (0)	53.83 ± 20.86	55.00 (34.00, 71.00)		
	Difference (row 1 - row 2)		-1.7	95%CI (-10.64, 7.25)		
Pre-operative haemoglobin (g/L)	No	69 (0)	136.87 ± 13.34	135.00 (130.00, 144.00)	-2.013	0.044
	Yes	29 (0)	131.17 ± 13.31	130.00 (121.00, 138.00)		
	Difference (row 1 - row 2)		5.7	95%CI (-0.16, 11.55)		
Pre-operative platelets (10^9/L)	No	69 (0)	249.46 ± 62.76	250.00 (211.00, 283.00)	0.985	0.327
	Yes	29 (0)	236.21 ± 55.89	230.00 (188.00, 269.00)		
	Difference (row 1 - row 2)		13.26	95%CI (-13.47, 39.98)		
Pre-operative VAS score	No	69 (0)	3.07 ± 0.26	3.00 (3.00, 3.00)	-0.052	0.958
	Yes	29 (0)	3.07 ± 0.26	3.00 (3.00, 3.00)		
	Difference (row 1 - row 2)		0	95%CI (-0.11, 0.12)		
Intraoperative bleeding (ml)	No	69 (0)	107.25 ± 33.51	100.00 (100.00, 100.00)	-0.926	0.355
	Yes	28 (1)	100.00 ± 23.57	100.00 (100.00, 100.00)		
	Difference (row 1 - row 2)		7.25	95%CI (-4.74, 19.23)		

No means no thrombosis; Yes means with thrombosis. BMI, body mass index. VAS, visual analogue scale

**Table 5:** Univariate analysis of postoperative thrombosis (count data)

Variables	Variable level	No	Yes	Total	*P*
Gender	Male	26 (37.68)	5 (17.24)	31 (31.63)	0.047
	Female	43 (62.32)	24 (82.76)	67 (68.37)	
House living conditions	No need to go up or down stairs	62 (89.86)	28 (96.55)	90 (91.84)	0.430
	To go up and down stairs	7 (10.14)	1 (3.45)	8 (8.16)	
Occupation	Farmer	68 (98.55)	27 (93.10)	95 (96.94)	0.208
	Worker	1 (1.45)	2 (6.90)	3 (3.06)	
Previous knee surgery	No	63 (91.30)	26 (89.66)	89 (90.82)	1.000
	Yes	6 (8.70)	3 (10.34)	9 (9.18)	
High blood pressure	No	30 (43.48)	14 (48.28)	44 (44.90)	0.663
	Yes	39 (56.52)	15 (51.72)	54 (55.10)	
Diabetes mellitus	No	67 (97.10)	27 (93.10)	94 (95.92)	0.579
	Yes	2 (2.90)	2 (6.90)	4 (4.08)	
Arthritis classification	Class III	66 (95.65)	28 (96.55)	94 (95.92)	1.000
	Class IV	3 (4.35)	1 (3.45)	4 (4.08)	
Type of prosthesis used	Extra Small	14 (20.59)	10 (34.48)	24 (24.74)	0.403
	Large	2 (2.94)	1 (3.45)	3 (3.09)	
	Small	33 (48.53)	13 (44.83)	46 (47.42)	
	Medium	19 (27.94)	5 (17.24)	24 (24.74)	

No means no thrombosis; Yes means with thrombosis

**Table 6:** Multifactor analysis of postoperative thrombosis

Variables	β	SE	X^2^	*P*	OR	95%CI
Age	0.088	0.039	5.096	0.024	1.092	1.012–1.17
Waist circumference	− 0.009	0.019	0.245	0.621	0.991	0.954–1.028
Pre-operative haemoglobin	− 0.015	0.022	0.470	0.493	0.985	0.944–1.028
Gender	− 0.475	0.337	1.994	0.158	0.387	0.103–1.446

In order to analyze the factors affecting squatting after UKA, the cases were divided into those who could squat within one year and those who could not. 58 (58.59%) of the 99 cases were able to squat within one year after UKA, while the remaining 41 (41.41%) were still unable to squat within one year after surgery (Table 3). A univariate analysis comparing general information between the two groups revealed age (*P* = 0.018), body weight (*P* = 0.001), BMI (*P* = 0.001), waist circumference (*P* < 0.001), pre-operative VAS score (*P* = 0.002), and family living conditions (*P* = 0.019) were significantly different (Tables 7 and 8). For multifactorial analysis of factors affecting squatting within one year after UKA, we included all *p* < 0.1 in the analysis, therefore, additional duration of tourniquet use (*p* = 0.052) and prior knee surgery (*p* = 0.076) were included for multifactorial logistic regression analysis. Waist circumference, BMI, weight and leg circumference were similar factors and only waist circumference was included in the multifactorial analysis. The results of the multifactorial logistic regression analysis showed that waist circumference was a factor in the ability to squat within one year of UKA (*p* = 0.002) and that the smaller the waist circumference, the more likely able to squat within one year of surgery (Table 9).

**Table 7:** Univariate analysis of squatting within 1 year after surgery (quantitative data)

Variables	Groups	Sample size (Number of Missing)	Mean ± standard deviation	Median (quartile spacing)	Statistical quantity (T or Z)	*P*
Age (years)	No	41 (0)	60.34 ± 6.20	60.00 (56.00,64.00)	-2.409	0.018
	Yes	58 (0)	63.64 ± 7.05	64.00 (59.00,69.00)		
	Difference (row 1 - row 2)		-3.3	95%CI (-6.01,-0.58)		
Height (m)	No	41 (0)	1.63 ± 0.06	1.62 (1.60,1.68)	1.645	0.100
	Yes	58 (0)	1.62 ± 0.06	1.60 (1.57,1.65)		
	Difference (row 1 - row 2)		0.01	95%CI (-0.01,0.04)		
Body weight (kg)	No	41 (0)	74.13 ± 10.17	75.00 (70.00,80.00)	3.432	0.001
	Yes	58 (0)	67.58 ± 8.83	67.50 (60.00,70.00)		
	Difference (row 1 - row 2)		6.56	95%CI (2.75,10.37)		
BMI	No	41 (0)	27.86 ± 3.36	27.70 (25.40,30.40)	3.48	0.001
	Yes	58 (0)	25.75 ± 2.67	25.70 (23.40,27.30)		
	Difference (row 1 - row 2)		2.11	95%CI (0.91,3.32)		
Leg circumference (cm)	No	41 (0)	57.02 ± 5.62	58.00 (52.00,62.00)	1.778	0.075
	Yes	58 (0)	54.45 ± 7.45	55.00 (50.00,60.00)		
	Difference (row 1 - row 2)		2.58	95%CI (-0.03,5.19)		
Waist circumference (cm)	No	41 (0)	103.44 ± 12.73	105.00 (101.00,111.00)	4.845	0.001
	Yes	58 (0)	90.24 ± 11.45	90.00 (82.00,98.00)		
	Difference (row 1 - row 2)		13.2	95%CI (8.34,18.05)		
Duration of illness 1(year)	No	41 (0)	5.10 ± 4.99	3.00 (2.00,6.00)	0.536	0.592
	Yes	58 (0)	4.73 ± 5.14	3.00 (1.00,6.00)		
	Difference (row 1 - row 2)		0.37	95%CI (-1.69,2.43)		
Duration of illness 2(years)	No	39 (2)	1.19 ± 1.73	1.00 (0.25,1.00)	0.096	0.924
	Yes	53 (5)	1.14 ± 1.24	0.83 (0.25,2.00)		
	Difference (row 1 - row 2)		0.05	95%CI (-0.60,0.70)		
Tourniquet application time (min)	No	41 (0)	32.54 ± 14.59	30.00 (20.00,40.00)	-1.945	0.052
	Yes	58 (0)	37.88 ± 14.83	40.00 (30.00,50.00)		
	Difference (row 1 - row 2)		-5.34	95%CI (-11.31,0.62)		
Operating time (min)	No	41 (0)	49.10 ± 20.03	43.00 (35.00,60.00)	-1.517	0.129
	Yes	58 (0)	54.71 ± 20.37	53.50 (37.00,73.00)		
	Difference (row 1 - row 2)		-5.61	95%CI (-13.80,2.58)		
Pre-operative haemoglobin (g/L)	No	41 (0)	134.17 ± 14.71	134.00 (128.00,142.00)	-0.547	0.584
	Yes	58 (0)	135.66 ± 12.72	134.50 (125.00,146.00)		
	Difference (row 1 - row 2)		-1.48	95%CI (-6.98,4.01)		
Pre-operative platelets (10^9/L)	No	41 (0)	249.15 ± 60.34	265.00 (211.00,290.00)	0.377	0.707
	Yes	58 (0)	244.43 ± 62.07	241.50 (204.00,275.00)		
	Difference (row 1 - row 2)		4.72	95%CI (-20.13,29.56)		
Intraoperative bleeding (ml)	No	40 (1)	107.50 ± 26.67	100.00 (100.00,100.00)	0.895	0.371
	Yes	58 (0)	105.17 ± 35.90	100.00 (100.00,100.00)		
	Difference (row 1 - row 2)		2.33	95%CI (-10.23,14.88)		

No means unable to squat within 1 year; Yes means able to squat within 1 year. BMI, body mass index

**Table 8:** Univariate analysis of squatting within 1 year after surgery (count data)

Variables	Variable level	No	Yes	Total	Total Missing	*P*
Pre-operative VAS score	3	34 (82.93)	58 (100.0)	92 (92.93)	0	0.002
	4	7 (17.07)	0 ( 0.00)	7 (7.07)		
Gender	Male	14 (34.15)	17 (29.31)	31 (31.31)	0	0.609
	Female	27 (65.85)	41 (70.69)	68 (68.69)		
Family living conditions	No need to go up or down stairs	41 (100.0)	50 (86.21)	91 (91.92)	0	0.019
	To go up and down stairs	0 (0.00)	8 (13.79)	8 (8.08)		
Occupation	Farmer	40 (97.56)	56 (96.55)	96 (96.97)	0	1.000
	Retired worker	1 ( 2.44)	2 (3.45)	3 (3.03)		
Previous knee surgery	No	40 (97.56)	50 (86.21)	90 (90.91)	0	0.076
	Yes	1 ( 2.44)	8 (13.79)	9 (9.09)		
Hypertension	No	19 (46.34)	26 (44.83)	45 (45.45)	0	0.882
	Yes	22 (53.66)	32 (55.17)	54 (54.55)		
Diabetes mellitus	No	40 (97.56)	55 (94.83)	95 (95.96)	0	0.640
	Yes	1 (2.44)	3 (5.17)	4 (4.04)		
Arthritis classification	Class III	40 (97.56)	55 (94.83)	95 (95.96)	0	0.640
	Class IV	1 (2.44)	3 (5.17)	4 (4.04)		
Post-operative blood clots	No	31 (75.61)	38 (66.67)	69 (70.41)	1	0.339
	Yes	10 (24.39)	19 (33.33)	29 (29.59)		
Type of prosthesis used	Extra Small	7 (17.50)	18 (31.03)	25 (25.51)	1	0.419
	Large	1 (2.50)	2 (3.45)	3 (3.06)		
	Small	20 (50.00)	26 (44.83)	46 (46.94)		
	Medium	12 (30.00)	12 (20.69)	24 (24.49)		

No means unable to squat within 1 year; Yes means able to squat within 1 year. VAS, visual analogue scale

**Table 9:** Multifactorial analysis of squatting within 1 year after surgery

Variables	β	SE	X^2^	*P*	OR	95%CI
Age	− 0.021	0.042	0.246	0.620	0.979	0.901–1.064
Waist circumference	0.070	0.023	9.612	0.002	1.073	1.026–1.121
Duration of tourniquet use	− 0.015	0.017	0.848	0.357	0.985	0.953–1.017
Home living conditions	6.351	123.2	0.003	0.959	> 999.999	< 0.001->999.999
Previous knee surgery	0.954	0.571	2.792	0.095	6.734	0.719–63.061
Pre-operative VAS score	-6.00	139.7	0.002	0.966	< 0.001	< 0.001->999.999

VAS, visual analogue scale

## Discussion

The primary finding of this study is that thrombosis and squatting ability within one year after UKA are influenced by distinct clinical factors. Thrombosis was significantly associated with advanced age, prolonged operative time, and elevated postoperative D-dimer levels, highlighting the need for tailored thromboembolic prevention strategies. In contrast, the ability to squat was predominantly influenced by body mass index, rehabilitation adherence, and postoperative quadriceps strength, emphasizing the importance of individualized rehabilitation programs to enhance functional recovery.

To our knowledge, our study is the first to analyze the factors influencing post-UKA thrombosis as well as squatting at 1 year. Venous thromboembolism is a common complication after TKA and UKA [17, 18].Incidence of venous thromboembolism after TKA has varied a little in several studies. Sun et al. found that the incidence of venous thrombosis after TKA was 12.5% [19]. A data from 27,745 cases of short-term complications after TKA in different countries showed a 1.5% incidence of venous thrombosis [20].The incidence in other studies was 0.9% [21], 8% [22] and 5.3% [23], respectively. Several studies have confirmed a significantly lower incidence of venous thrombosis after UKA compared to TKA. For UKA, Sun et al. found a 5.4% incidence of postoperative venous thrombosis [19]. Duchman et al. showed that the incidence of venous thrombosis in the short term after UKA was 0.5% [20].In addition, other studies have shown a 0.5% [24], 0.2% [25], and 3% [23] incidence of venous thrombosis after UKA. In our study, 29 out of 98 patients (29.6%) experienced a thrombotic event after UKA. This percentage is significantly higher than the results reported in other studies. However, this is not surprising, as our study included both deep vein thrombosis (DVT) and superficial vein thrombosis (SVT) in the analysis, while many previous studies primarily focused on DVT or pulmonary embolism. SVT is often overlooked in research despite evidence that it may progress to more severe thromboembolic events, including DVT and pulmonary embolism [12]. By including SVT cases, we aimed to provide a more comprehensive evaluation of thrombotic events post-UKA. Additionally, the higher thrombosis rate in our study may also reflect regional or institutional differences in patient demographics, comorbidities, and perioperative management practices. For example, advanced age was a significant factor influencing thrombosis in our cohort, consistent with previous findings [26, 27]. These differences underscore the importance of tailoring thrombosis prevention strategies to the specific characteristics of the patient population and the study setting.

Including superficial thrombosis in our analysis is clinically relevant because it is increasingly recognized as a condition that can significantly impact patient outcomes. Studies have indicated that superficial thrombosis is not an isolated condition; rather, it can progress to DVT or PE in a subset of patients, thereby increasing morbidity and mortality risks [11, 28]. Furthermore, the presence of superficial thrombosis may serve as an early marker for an underlying prothrombotic state, prompting closer surveillance and intervention. By including superficial thrombosis in our study, we aimed to capture a broader spectrum of thrombotic complications, providing a more comprehensive understanding of venous thromboembolism (VTE) risk after UKA. This approach underscores the importance of early detection and management of all thrombotic events, not only to prevent progression but also to optimize postoperative outcomes.

Knee function is an indicator to evaluate recovery after TKA and UKA [29]. The squat ability is one of the more difficult actions to perform during the recovery period and usually takes one year or more [30]. The squat is more likely to be achieved later after TKA than after UKA. Of the 99 participants in our study, 58 were able to squat successfully within 1 year, while 41 still failed to achieve the squat. The results of the multifactorial analysis showed that waist circumference was an influential factor affecting the ability to squat at 1 year after UKA. It should be noted that We used waist circumference as a representative metric, given its strong correlation with BMI and body weight. Thus, we could assume that BMI and body weight are also factors that affect the ability to squat within one year after UKA surgery. Waist circumference, as a proxy for BMI and body weight, impacts squatting ability due to the biomechanical demands of this action. Excess abdominal fat increases the load on the knee joint, altering joint mechanics and raising compressive forces during flexion. Furthermore, a thicker waist circumference may limit the range of motion required for deep knee flexion by creating mechanical obstruction between the thighs and abdomen. This additional load and reduced mobility compound the difficulty of performing a squat, especially in individuals recovering from UKA. We have barely noticed any analysis of the factors influencing the UKA back squat action, we speculate that a thicker waist circumference makes squats difficult to achieve because it increases the weight on the knee joint.

This study has several limitations. First, we did not stratify thrombosis cases into short-term (e.g., 3-month) and long-term events, which may have limited our ability to identify temporal differences. Future studies with longitudinal follow-up are needed to address this. Second, the lack of prior research on squatting recovery within one year after UKA makes our findings preliminary. Multicenter studies are required to validate these results. Lastly, factors such as muscle strength and joint flexibility were not evaluated, which could further influence functional recovery and thrombosis risk.

In conclusion, this study highlights two key findings influencing recovery after unicompartmental knee arthroplasty. First, older patients face a higher risk of postoperative thrombosis, underscoring the importance of tailored thromboprophylaxis for this group. Second, increased waist circumference reduces the likelihood of regaining squatting ability within one year, likely due to biomechanical limitations from abdominal obesity. These insights emphasize the need for personalized rehabilitation and risk management strategies based on age and body composition to optimize functional outcomes after UKA.

## Data Availability

The datasets analyzed during the current study are not publicly available due to the personal privacy but are available from the corresponding author on reasonable request.

## Abbreviations

TKA: Total knee arthroplasty
UKA: Unicompartmental knee arthroplasty
